# Investigation and Optimization of a Line-Locked Quartz Enhanced Spectrophone for Rapid Carbon Dioxide Measurement

**DOI:** 10.3390/s21155225

**Published:** 2021-08-02

**Authors:** Hui Zhang, Wenling Jin, Mengpeng Hu, Mai Hu, Jingqiu Liang, Qiang Wang

**Affiliations:** 1 State Key Laboratory of Applied Optics, Changchun Institute of Optics, Fine Mechanics and Physics, Chinese Academy of Sciences, Changchun 130033, China; zhanghui195@mails.ucas.ac.cn (H.Z.); humengpeng19@mails.ucas.ac.cn (Mengpeng Hu); humai@ciomp.ac.cn (Mai Hu); liangjq@ciomp.ac.cn (J.L.); 2 University of Chinese Academy of Sciences, Beijing 100049, China; 3 National Key Laboratory of Science and Technology on Tunable Laser, Harbin Institute of Technology, Harbin 150001, China; 21B321011@stu.hit.edu.cn

**Keywords:** quartz enhanced photoacoustic spectroscopy, carbon dioxide, custom quartz tuning fork, wavelength locking, laser spectroscopy

## Abstract

We have developed a rapid quartz enhanced spectrophone for carbon dioxide (CO_2_) measurement, in which the laser wavelength was tightly locked to a CO_2_ absorption line and a custom quartz tuning fork (QTF) operating at 12.5 kHz was employed. The intrinsic QTF oscillation-limited response time, as well as the optimal feedback interval, was experimentally investigated. By tightly locking the laser to the R(16) transition of CO_2_, we obtained a stable laser operation with its center wavelength variation kept within 0.0002 cm^−1^, merely three times the laser linewidth. The reported CO_2_ sensor achieved a detection limit of 7 ppm, corresponding to a normalized noise equivalent absorption coefficient (NNEA) of 4.7 × 10^−9^ W·cm^−1^·Hz^−1/2^, at a response time of 0.5 s. The detection limit can be further improved to 0.45 ppm at an integration time of 270 s, illustrating a good system stability. This spectrophone enables the realization of compact and fast-response gas sensors for many scenarios, where CO_2_ concentration from sub-ppm to hundreds of thousands of ppm is expected.

## 1. Introduction

Carbon dioxide (CO_2_) is an important component of the atmosphere that protects life on Earth via photosynthesis and heat preservation. However, its adverse impact, mainly the greenhouse effect, is emerging with massive emissions from increasingly extensive and intensive human activities in industrial, agricultural, and ecological fields [1]. In addition to our living environment, biological CO_2_ functions as a valuable biomarker for human disease predictions such as *Helicobacter pylori* infection, lung lesions, and liver malfunctions [2]. Moreover, CO_2_ abundance and its variation tendency are critical in many other applications, including combustion analysis, industrial process control, and deep-sea exploration [3,4,5]. Therefore, CO_2_ sensors with fast response, high sensitivity, and wide dynamic range are desirable across a broad range of fields.

Currently, commercially available instruments for CO_2_ detection include gas chromatography systems, electrochemical sensors, non-dispersing infrared analyzers, and infrared spectrometers. Suffering from bulky size, short service life and even poor selectivity, most of them are not well suited to scenarios where real-time and in situ CO_2_ measurement is highly needed. In contrast, the development of photoacoustic spectroscopy (PAS) is filling this gap [6,7,8]. PAS relies on the detection of acoustic waves induced by the process of optical heat deposition and thermal expansion. In particular, quartz-enhanced photoacoustic spectroscopy (QEPAS), exploiting a tiny quartz tuning fork (QTF) as an acoustic transducer, is one of the most attractive techniques, with high selectivity, high sensitivity, good immunity to environmental acoustic noise, and ultra-low gas consumption [9,10]. Since its invention by Kosterev et al. in 2002 [11], QEPAS has been demonstrated for detection of numerous organic and inorganic trace gases, including precise CO_2_ measurements by spectroscopy analysis at trace level, molecular relaxation investigations and interference studies on other gaseous species [12,13,14].

The commonly used standard QTFs were optimized for timing purposes, operating at ~32.7 kHz, instead of spectroscopic applications. On the other side, the energy transfer of CO_2_ cannot follow the fast molecular vibration excitation due to its relatively long relaxation time constant [13]. Thus, an inadequately generated acoustic wave results in a weaker signal. To date, most QEPAS CO_2_ sensors have a concentration detection limit of tens to hundreds of ppm, especially when the in situ excitation power is limited to merely mW level [15,16,17,18]. Following the radiation-to-sound conversion efficiency *ε* of photoacoustic process, i.e., *ε* = [1 + (2π*f*_0_*τ*)^2^]^–1/2^, where *τ* is the relaxation time [19], the CO_2_ QEPAS signal can benefit from a QTF with lower resonant frequency *f*_0_. Since the breakthrough of custom-made QTFs in 2013, QEPAS sensors have been capable of operating at a frequency much more suitable to low-relaxation-rate gas species [20,21].

Generally, QEPAS spectrophones are based on 2*f* wavelength modulation spectroscopy. A sub-Hertz wavelength scanning, along with a modulation at half of the QTF resonance frequency, is used to plot the 2*f* spectrum, whose maximum value always appears at the absorption line center, leading to a simple model to diagnose the analyte. However, the response time of scanning-assisted QEPAS can be extended up to tens of seconds due to its high-quality factor Q (10,000 to 13,000) [22]. That is still too slow to perform rapid gas measurements, especially when the information needs to be captured in real time. This issue could be mitigated if the absorption feature can be targeted without scanning. To prevent the laser frequency drift from the fluctuation of laser operation temperature and current, several rugged methods have been commonly applied to gas sensing, such as real-time wavelength stabilization using odd harmonics as the error signal [23,24] and quasi wavelength locking with a regular calibration by referring to the second harmonic maximum [25].

In this work, a LabVIEW-based servo loop was developed to perform the line-locked process, in which the laser wavelength was tightly locked to the target transition line of CO_2_. A sensitive and rapid CO_2_ sensor, combining the line-locked process and a custom QTF with a low resonant frequency of 12.5 kHz, was demonstrated. Averaging filtering was then fully exploited on data processing for lower system noise without sacrificing the response time. Response time limitation and the optimal feedback interval were investigated by experimental measurements. Ultimately, the sensor was validated to measure the ambient background and exhaled CO_2_ concentration, in a field application.

## 2. Sensor Configuration and Line-Locked Process

**Figure 1:**
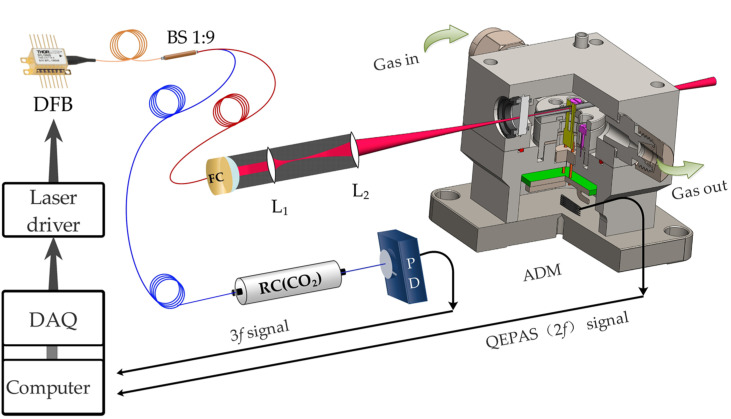
Line-locked quartz enhanced spectrophone. BS: beam splitter; FC: fiber collimator; L_1_ and L_2_: mode-matching lens; RC: reference chamber; PD: photoelectric detector; DAQ: data acquisition card; ADM: acoustic detection module.

**Figure A1:**
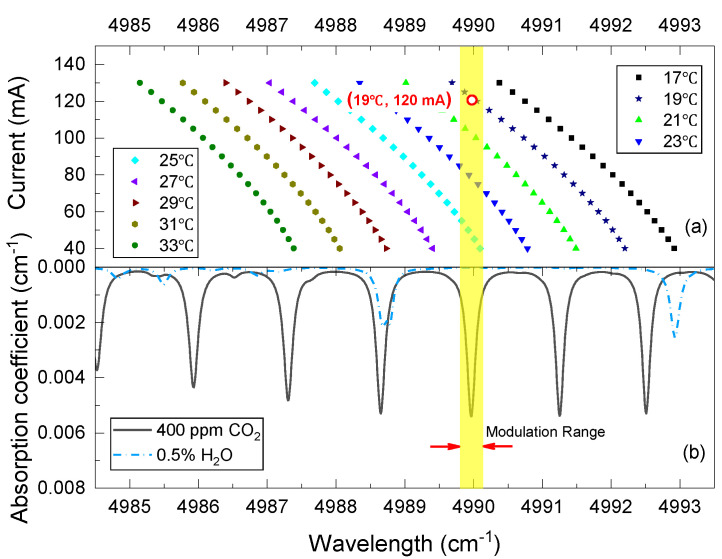
Optimization of driving temperature and bias current. (**a**) DFB laser specification, (**b**) absorption coefficient simulation for 400 ppm CO_2_ and 0.5% H_2_O at 296 K and 1 bar. Driving temperature and bias current were selected as 19 °C and 120 mA, respectively, to target the R(16) line of CO_2_ at 4989.97 cm^−1^ [32]. The yellow shadow depicts the modulation range of ~0.31 cm^−1^.

A line-locked quartz enhanced spectrophone system was built with the configuration shown in Figure 1. The sensing system consists of a wavelength locking part and a gas sensing part. A 2.004 μm DFB laser diode (KELD1G5BAAA, NTT Electronics, Yokohama-shi, Japan) is driven by a LabVIEW-based electrical control unit and a low noise laser driver (LDC501, Stanford Research Systems, Sunnyvale, CA, USA). After careful laser characterization, the driving temperature and bias current are selected as 19 °C and 120 mA, respectively. Laser radiation thus can be obtained to exploit the R(16) line of CO_2_ with less spectral overlap interference and high laser power (see Appendix A Figure A1). An acoustic detection module (ADM01, Thorlabs, Newton, NJ, USA) is used the perform CO_2_ measurement, this ADM consists of a custom QTF as the acoustic transducer, two acoustic resonators for acoustic amplification, and two wedged BaF_2_ windows for optical access. A small fraction of the DFB laser radiation, separated by a 1:9 fiber splitter, interrogates a pigtailed CO_2_ chamber (length 6 cm; pressure 50 Torr) for wavelength locking. The rest of the laser radiation (calibrated as 5 mW) propagates through the on-beam acoustic resonators (inner diameter: 1.6 mm; length: 12.4 mm) and the custom QTF (prong space: 800 μm) successively. Two mode-matching lenses, L_1_ and L_2_, allow adequate focalization of the excitation beam down to ~200 μm between QTF prongs without hitting them. With the laser modulated at half of the QTF resonance frequency *f*_0_, a photoacoustic wave is excited at the presence of the analyte. A trans-impedance preamplifier with a feedback resistance of 10 MΩ is used for signal amplification prior to demodulation by a lock-in amplifier (MFLI, Zurich Instruments, Zurich, Switzerland).

**Figure 2:**
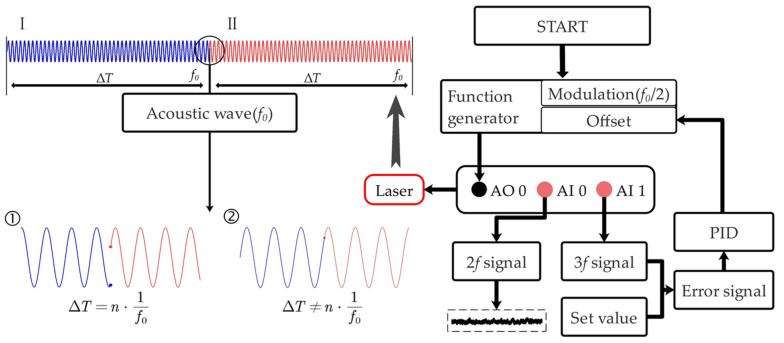
**Right part:** Flow chart for line-locked process based on a LabVIEW platform. Offset: a preset bias current corresponding to a wavelength near the absorption line; *f*_0_: resonant frequency of the custom QTF. **Left part:** Feedback interval Δ*T* should be selected as an integer multiple of the QTF oscillation period, thus phase matching between successive modulation periods I and II can be maintained for continuous measurement without counteraction.

To perform tight wavelength locking to the absorption line, we developed a LabVIEW-based software platform with the flow chart shown in Figure 2. Usually, for wavelength modulation spectroscopy, the odd harmonics have centrosymmetric profiles about the absorption line center [23,24,25]. Hence, the third harmonic (3*f* signal) is used to generate an error signal for the following line-locked process. Firstly, a preset bias current, as well as the modulation signal at *f*_0_/2, is applied via a digital-analog converter (i.e., AO0) of the DAQ card (USB-6356, National Instruments, Austin, TX, USA), generating a radiant wavelength near the absorption line. Secondly, the CO_2_ absorption information inside the reference chamber is captured via the analog-digital converter (i.e., AI1) and then is demodulated as the 3*f* signal using a digital lock-in amplifier. Thirdly, a PID subprogram is employed to make the 3*f* signal closer to the set value (zero in this paper) by iterating the bias current. Thus, the CO_2_ measurement is conducted with the photoacoustic signal captured (via AI0) and averaged during the feedback interval.

Note that, the feedback interval (Δ*T*) cannot be discretionarily selected as it would have a significant influence on the tuning fork response. As shown in Figure 2, if the ending of modulation period I is not connected with the beginning of the successive modulation period II, the phase mismatch will break the energy accumulation process of QTF, leading to a weakened photoacoustic signal. Our hypothesis is confirmed by a detailed feedback interval investigation, which can be found in Experimental Results and Discussion section below. Thus, in the implementation of line-locked QEPAS, phase matching between successive modulation periods should be considered in selecting the feedback interval, which should be an integer multiple (*n*) of the QTF oscillation period as:

(1) $$∆T=n/f_{0}$$

## 3. Experimental Results and Discussion

### 3.1. Response Time of the QTF Spectrophone

**Figure 3:**
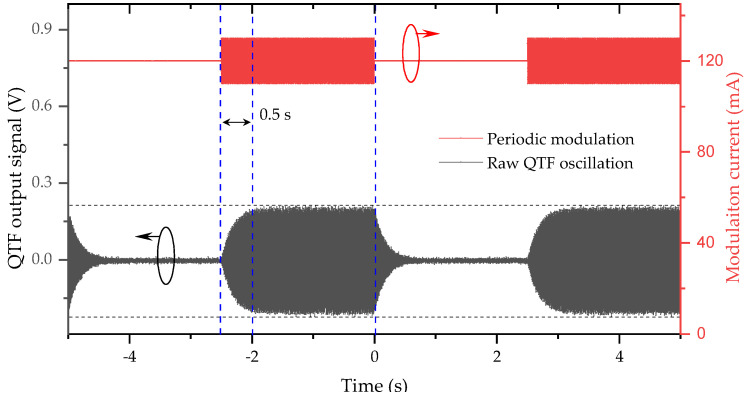
Real-time QTF oscillation response to a periodic stimulation. The stimulation was generated by applying driving current with and without modulation on the laser, successively. The repeat frequency was 0.2 Hz, in which the 2.5-s period without modulation was long enough for oscillation release prior to the following stimulation. Response time, from 0 to 100% oscillation, was determined to be 0.5 s.

**Figure A2:**
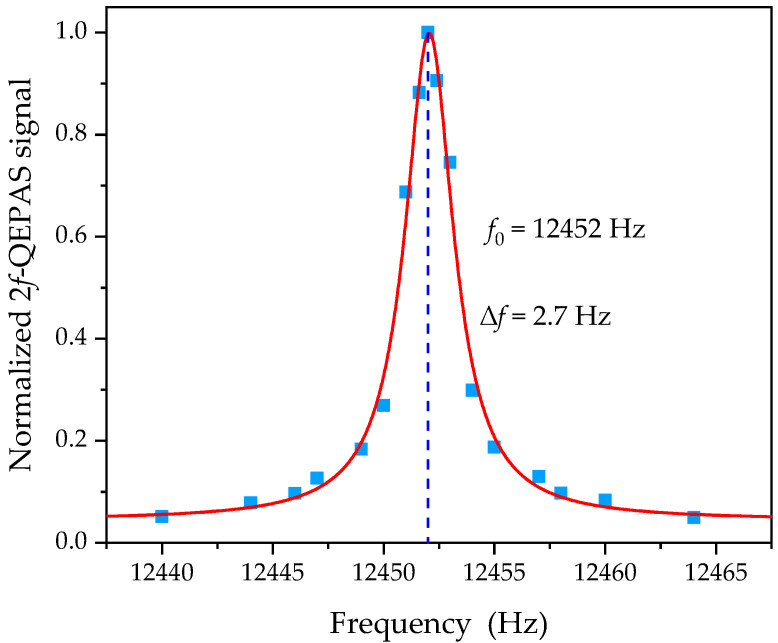
Frequency response characteristics of the custom QTF at 1 bar and 296 ± 2 K. The investigation was performed with 5000-ppm CO_2_/N_2_ mixture filled into the ADM.

QEPAS acoustic wave detection strongly relies on sufficient energy accumulation in a QTF, which, in turn, limits the response time of the spectrophone. The effective QEPAS response to CO_2_ was experimentally determined by real-time monitoring of the QTF oscillation curve, in which acoustic stimulation was generated after sufficient QTF oscillation release (2.5 s). CO_2_ (1%) was filled into the ADM and the radiation wavelength was turned to the absorption line prior to the response time investigation. Figure 3 depicts the measured raw data of QTF oscillation response to the periodic stimulation. Based on the time it takes for signal amplitude increases from zero to a plateau level, the response time is determined to be 0.5 s, while the time it takes to reach 90% of the final level is 0.365 s, that is consistent with the theoretical model of τ = Q/*f*_0_ [11,26] using the calibrated quality factor Q and resonant frequency *f*_0_ (see Appendix B Figure A2). Note that Q and *f*_0_ depend on the operation temperature and gas pressure [27], all the following experiments are performed under room temperature and 1 bar.

### 3.2. Feedback Interval Determination

**Figure 4:**
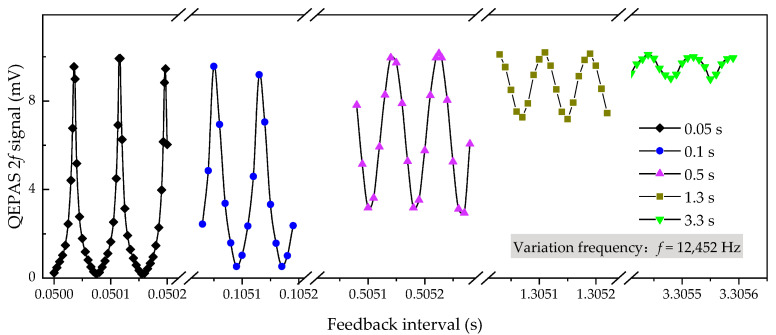
QEPAS response curves to feedback intervals around 0.05 s, 0.1 s, 0.5 s, 1.3 s, and 3.3 s respectively. The experiment was performed with 1000-ppm CO_2_ enclosed inside the ADM.

The photoacoustic signal response to feedback interval was investigated to optimize the CO_2_ measurement with maximum amplitude. Sample gas with 1000-ppm CO_2_ diluted in N_2_ was sealed in the ADM and a modulation current (frequency: 6226 Hz; amplitude: 9.4 mA) was added on an iterated bias current. Figure 4 depicts the QEPAS signals with periodic variations around typical feedback intervals of 0.05 s, 0.1 s, 0.5 s, 1.3 s, and 3.3 s. As expected, the variation frequency, which is obtained by averaging the five oscillation periods, equals the resonant frequency of the custom QTF.

Interestingly, stronger variation amplitude exists around a shorter feedback interval. This is probably due to the short energy accumulate and limited accumulated energy can be easily counteracted down to almost zero by the successive phase-mismatched acoustic source. This issue can be mitigated with a longer feedback interval, e.g., oscillation amplitudes around 1.3 s and 3.3 s are alleviated and will become imperceptible over an interval over 8 s. Again, the hypothesis on phase matching in reasonable feedback interval selection has been confirmed. Although similar maxima, compared with feedback intervals of 0.5 s or longer, can be obtained at about 0.05 s and 0.1 s without interrupting energy accumulation, sharper oscillations would make the spectrophone need a rather stable electronic control unit. Any small fluctuations (e.g., clock jitter, time difference of data processing for each feedback interval etc.) would lead to a misinformation. Considering the response time of the QTF spectrophone shown in Figure 3 as well as the feedback interval investigation, we selected a feedback interval of 0.50514 s to perform tight wavelength locking for the following experiments, while maintaining sufficient energy accumulation.

### 3.3. Line-Locked Wavelength Stabilization

**Figure 5:**
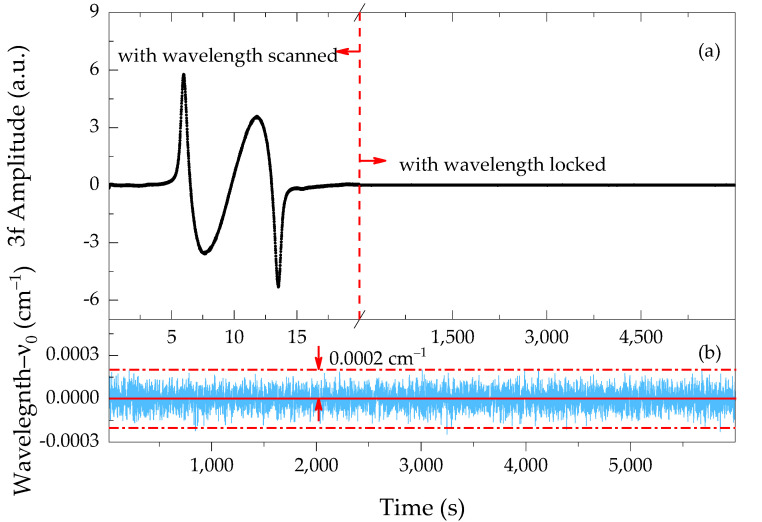
(**a**) 3*f* amplitude of reference chamber under both laser wavelength slowly scanned mode and tightly locked mode. (**b**) Stable laser operation with its wavelength variation kept within 0.0002 cm^−1^ over 6000 s, meanwhile, a feedback interval of 0.50514 s was chosen.

The DFB laser wavelength was firstly scanned across the CO_2_ spectrum by superposing a 0.05 Hz ramp on a 6226 Hz sinusoidal modulation. The preset bias current, corresponding to the absorption line center ν_0_, was then figured out for the following line-locked process. The 3*f* component of the absorption feature was used as the error signal to compensate for the laser wavelength drift. With the PID feedback control started, as shown in Figure 5, the 3*f* reaches an approximately constant value with a small variation around zero for hours, meaning real-time wavelength locking to the absorption line during continuous operation. One might argue that when working at atmospheric pressure (ADM, 760 Torr) there is a shift of the CO_2_ line, with respect to the reference chamber (50 Torr). In this case, the shift is about 150 MHz [28], only 3% of the CO_2_ linewidth (4.4 GHz at 760 Torr). Its influence on the signal amplitude is evaluated to be within 0.5%. Besides, a narrower linewidth at low pressure can benefit the wavelength locking because the error signal is more sensitive to the laser frequency drift. The wavelength stabilization performance is evaluated by comparing the 3*f* amplitude fluctuation under line-locked operation with the scanning spectrum (i.e., wavelength scanned part in Figure 5a). The 3*f* amplitude over 6000 s is shown in Figure 5b with the wavenumber deviations evaluated to be within 0.0002 cm^−1^. Therefore, an improvement in stability, accuracy, and response time can be expected for QEPAS spectrophones.

### 3.4. Sensor Performance

**Figure 6:**
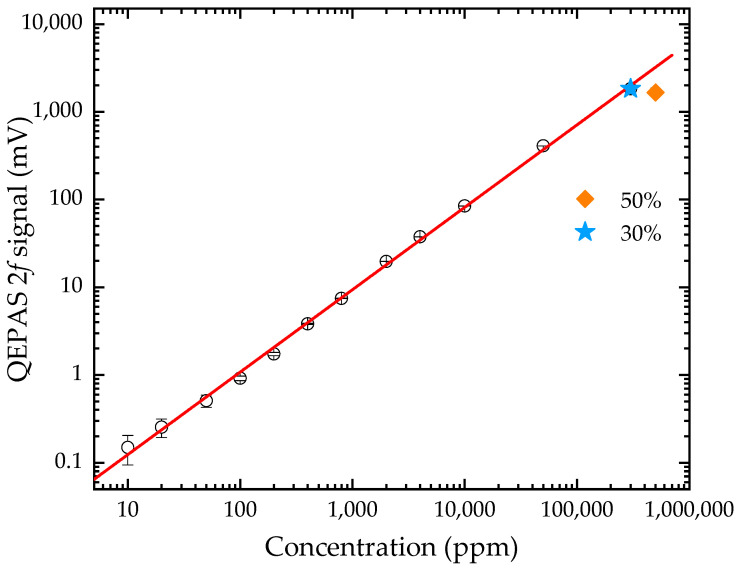
Linearity of the QEPAS 2*f* signal as a function of CO_2_ concentration at 1 bar and 296 ± 2 K. The linear fitting yields an R-square value of 0.9985 from 10 ppm to 30%.

**Figure A3:**
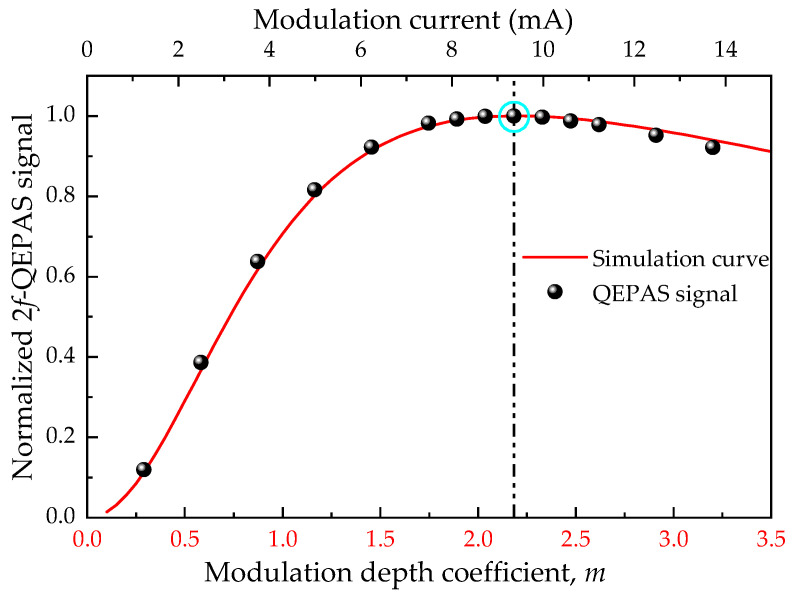
Normalized 2*f* QEPAS signal as a function of modulation current and simulated QEPAS response as a function of modulation depth coefficient *m*. In this case, the modulation current of 9.4 mA corresponds to the maximum 2*f*-QEPAS signal at *m* = 2.2.

After completing the construction of the experimental system, we first optimized the laser wavelength modulation depth coefficient (i.e., m = α/Δν, where α is modulation depth and Δν is the HWHM of the absorption spectrum) to improve the 2*f* QEPAS signal amplitude (see Appendix C Figure A3). With the laser wavelength tightly locked to the absorption line, the sensor performance was further investigated at 1 bar and 296 ± 2 K. CO_2_/N_2_ mixtures were uniformly pumped through the ADM at a flow rate of 200 mL/min. The mixtures were prepared by diluting certified 1% and 100% CO_2_ with pure nitrogen (purity 99.999%) using a commercial gas mixer. The sensor response is illustrated in Figure 6 by plotting the photoacoustic 2*f* amplitude as a function of CO_2_ concentration, ranging from 10 ppm to 50%. The vertical error bars take into account the uncertainty of the measured signal amplitude (i.e., the standard deviation of measurement over 30 s). A linear fitting is performed to the measured data and an R-square value of 0.9985 is obtained, indicating a good linear response to the CO_2_ concentration. Above a CO_2_ concentration of 30%, the acoustic signal begins to decline. It is probably caused by the significant variation of gas components, which could change the QTF resonant frequency by a higher gas density, the optimal resonant tube length by a lower sound velocity, or the modulation depth coefficient by a broadened HWHM.

**Figure 7:**
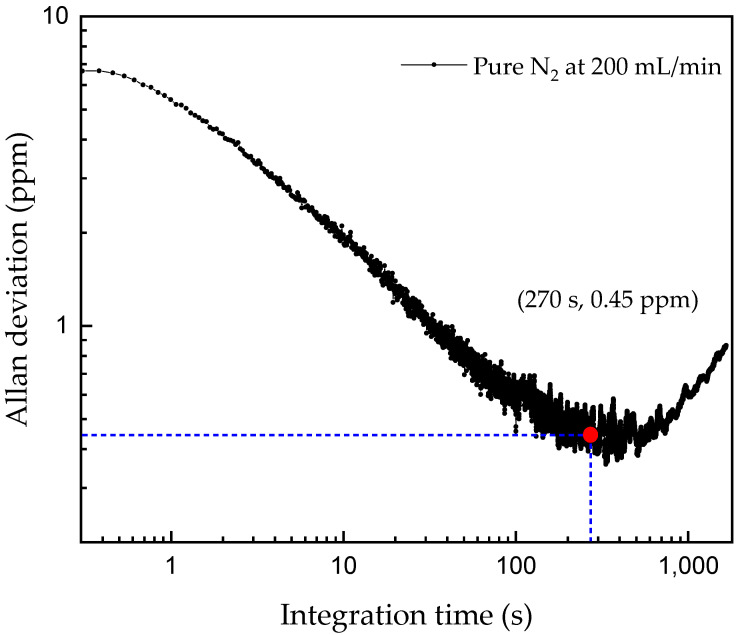
Allan deviation plot in ppm of the quartz enhanced spectrophone as a function of integration time. In the evaluation, basic parameters are analysis time, 140 min; detection bandwidth, 1 Hz; flow rate, 200 mL/min.

To evaluate the achievable minimum detection limit of the current QEPAS spectrophone, we performed an Allan deviation analysis, measuring and averaging the photoacoustic signal of continuous dry nitrogen at a flow rate of 200 mL/min. Meanwhile, the laser wavelength was locked to the absorption line of CO_2_. The Allan deviation plotted in Figure 7 reports a detection limit of 7 ppm at an integration time of ~0.5 s. The corresponding normalized noise equivalent absorption coefficient (NNEA) is calculated to be 4.7 × 10^−9^ W·cm^−1^·Hz^−1/2^. Furthermore, the detection limit can be improved to 0.45 ppm at an integration time of 270 s. This minimum detection limit together with the upper detection concentration of 30% in Figure 6 determines the linear dynamic range, i.e., 6.7 × 10^5^.

### 3.5. Field Applications for Atmospheric and Exhaled CO_2_ Measurement

**Figure 8:**
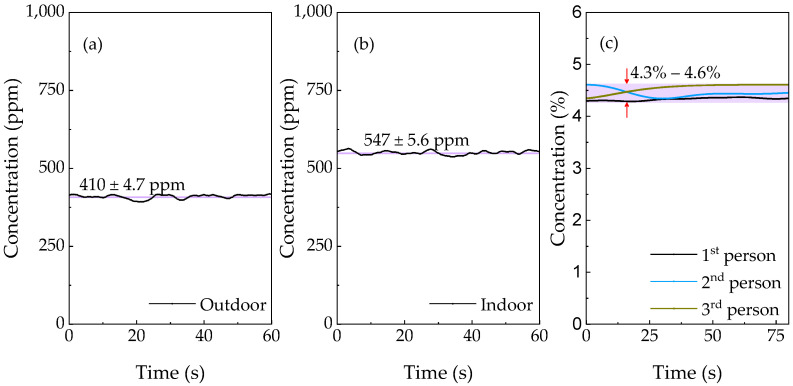
CO_2_ measurements of (**a**) outdoor and (**b**) indoor air, and (**c**) expirations of three people.

The field performance of the spectrophone was evaluated by measuring the CO_2_ level in air (both indoors and outdoors) and exhalations of three researchers, successively. The gas samples were fully dehumidified before each measurement inside the ADM, otherwise CO_2_ molecular relaxation would be accelerated by the presence of humidity in the gas [13,29], leading to enhanced photoacoustic amplitude, however, at the expense of sensing reliability under an unstable water vapor concentration. The CO_2_ concentrations are determined using the calibration curve in Figure 6 with the results depicted in Figure 8. The observed outdoor CO_2_ concentration, 410 ± 4.7 ppm, is in good agreement with global greenhouse gas monitoring [30]. While the indoor CO_2_ concentration, 547 ± 4.6 ppm is a bit higher, mainly due to the contribution by people working in the laboratory. The observed exhaled CO_2_ concentrations of three people vary from 4.3 to 4.6%, which lie in a healthy and common range according to a number of breath analysis studies, reviewed in [31].

## 4. Conclusions

In summary, we have realized a quartz-enhanced spectrophone for rapid CO_2_ measurement. A custom QTF with a low resonant frequency of 12.5 kHz was used to increase the QEPAS response to the slow-relaxing CO_2_. A LabVIEW-based servo loop was developed for stable laser operation with its center wavelength variation kept within 0.0002 cm^−1^. Phase match between successive feedback intervals, as well as the response time limitation of the QTF, was experimentally investigated. Thus, the response time of the QEPAS sensor was enhanced to ~0.5 s for continuous CO_2_ measurements while maintaining sufficient energy accumulation. Compared with several typical QEPAS-based CO_2_ measurements, which achieved a detection limit of tens to hundreds ppm [16,18,26], a better detection limit of 7 ppm was achieved, notably with a much faster response time. The system stability was evaluated by performing Allan deviation analysis, achieving a minimum detection limit of 0.45 ppm at an integration time of 270 s. Besides, a linear dynamic range of 6 × 10^5^ was obtained and the implementations for atmospheric and exhaled CO_2_ measurements, in a field application, were demonstrated. Future work to investigate the details of H_2_O influence on CO_2_ measurement with a humidity range from 0 to saturation is planned. The real-time correction will be performed by monitoring H_2_O concentrations to fully explore the capacity of this integrated quartz enhanced spectrophone for in situ and rapid CO_2_ measurements in many different fields.

## Data Availability

The data that support the plots within this paper are available from the corresponding author on request basis.

## Appendix A

The laser characteristic of a commercial DFB laser near 2.004 μm is shown in Figure A1a. A spectral range of 4985–4993 cm^−1^ can be accessed by adjusting the laser temperature (17–33 °C) and the injection current (40–140 mA). As depicted in Figure A1b, the absorption coefficient of 400 ppm CO_2_ and 0.5% H_2_O at 296 K and 1 bar are interrogated based on the HITRAN database. Bias current of 120 mA and temperature of 19 °C (labeled as a red circle) were selected to drive this DFB laser, and a modulation range of ~0.31 cm^−1^ (labeled as yellow shadow) was applied, as well. Therefore, the R(16) line of CO_2_ with a relatively strong intensity of 1.319 × 10^−21^ cm^−1^·(mol·cm^−2^)^–1^ can be interrogated with less spectral overlap interference, and be illuminated by a maximum laser power of about 5 mW, which was calibrated by a power meter after the ADM.

## Appendix B

The response curve of the custom QTF was characterized to determine the wavelength modulation frequency. CO_2_ sample with a concentration of 5000 ppm was pumped into the acoustic detection module (ADM). With the laser wavelength locked to the absorption line, modulation frequency was scanned across half of the expected resonant frequency. The 2*f* signal data were collected accordingly with the results shown in Figure A2. By performing a Lorentz fitting, the resonant frequency and full width at half-maximum (FWHM) of the resonance curve are calibrated as 12.452 kHz and 2.7 Hz, respectively, which yields Q = 4600. Theoretical intrinsic QTF oscillation limited response time can be hence calculated as 370 ms when the spectrophone is operated at 1 bar and 296 ± 2 K.

## Appendix C

The QEPAS spectrophone response to different modulation currents at a constant CO_2_ concentration of 1000 ppm (1 bar, 296 ± 2 K) is depicted in Figure A3. Photoacoustic signal amplitude increases with the modulation amplitude, but when the modulation current is higher than 9.4 mA, no further increase can be observed. The QEPAS response is consistent with the simulation result plotted as a red line and the optimized modulation current corresponds to a wavelength modulation depth coefficient of 2.2.
